# Oral health and self-rated health in community-dwelling older adults in Colombia

**DOI:** 10.1186/s12903-023-03401-4

**Published:** 2023-10-20

**Authors:** Luis Carlos Venegas-Sanabria, María Manuela Moreno-Echeverry, Miguel German Borda, Diego Andrés Chavarro-Carvajal, Carlos Alberto Cano-Gutierrez

**Affiliations:** 1 Instituto Rosarista para el Estudio del Envejecimiento y la Longevidad (IREEL), Escuela de Medicina Y Ciencias de La Salud, Universidad del Rosario, Bogotá, Colombia; 2 Centro de Investigaciones de Méderi, Hospital Universitario Mayor – Méderi, Bogotá, Colombia; 3 Unidad de Geriatría, Hospital Universitario San Ignacio, Bogotá, Colombia; 4 Instituto de Envejecimiento, Pontificia Universidad Javeriana, Bogotá, Colombia; 5 Semillero de Neurociencias Y Envejecimiento, Instituto de Envejecimiento, Pontificia Universidad Javeriana, Bogotá, Colombia; 6 Centre for Age-Related Medicine (SESAM), Stavanger University Hospital, Stavanger, Norway; 7 Faculty of Health Sciences, University of Stavanger, Stavanger, Norway

**Keywords:** Oral hygiene, Quality of life, Health status, Geriatric assessment, Aged

## Abstract

**Background:**

The relationship between oral health and specific health conditions, such as cardiovascular disease or cognitive impairment, has been extensively studied. However, the effect of oral health status on self-rated health has not been assessed. This could be relevant in older people considering that poor self-rated health status and oral diseases are highly prevalent in this population. The aim of this study was to determine the association between different parameters of oral health and self-rated health status (SRHS) in Colombian community-dwelling older adults.

**Methods:**

This is a secondary analysis of the SABE-Colombia study performed in 2015. The dependent variable was defined as the SRHS status assessed by the question "Compared with other people, your age: Do you consider your health status to be better, equal, or worse?” We considered four independent variables: total edentulism considering the high prevalence in older people, the GOHAI score to assess self-rated oral health, and the use of fixed and removable dental prostheses as potential modifiers of oral health. An adjusted ordinal logistic regression was performed by each independent variable.

**Results:**

After the exclusion of missing data, 17,945 persons were included in the final analysis. A total of 10.6% reported worse SRHS, 37.6% reported equal SRHS, and 51.6% reported better SRHS. The worse SRHS group was older and had a higher proportion of dependence, cognitive impairment, and depressive symptoms. The frequency of total edentulism and the lower mean score of GOHAI were significant in the worse SHRS group. An ordinal logistic regression for each independent variable was performed, finding that edentulism increases the probability of worse SHRS, while the GOHAI and use of removable or fixed dental prostheses increase the probability of better SRHS.

**Conclusion:**

We found an association between total edentulism, GOHAI Index, the use of dental prostheses (both removable and fixed), and self-rated health status, showing the relevance of oral health status to self-rated health status independent of comorbidities and geriatric syndromes. This result supports the inclusion of oral health evaluation in comprehensive geriatric assessment.

## Introduction

Due to the demographic transition and current advances in medicine, the population of older people is steadily increasing [1]. Consequently, chronic pathologies have risen in prevalence, a situation that carries an increase in functional impairment and the need for caregivers. This situation forces better health services and public policies. However, together with the rise of chronic pathologies, the prevalence of other conditions that traditionally have been incorrectly considered normal in older adults is also increasing [2]. Among them, poor oral health and lost teeth have been shown to compromise the overall health and quality of life of adults and, most especially, older adults [3, 4].

The prevalence of edentulism varies according to the region. In Canada, the overall rate of edentulism was between 6.4% and 21.7% of older adults between 65 and 79 years old, and this prevalence is higher in low- and middle-income countries (29% for older adults older than 80 years old in Indonesia) [5]. The importance of edentulism lies in that it affects the well-being of individuals, increases the risk of infections, alters the deglutition process, and generates speech problems, social isolation, and malnutrition, just to mention a few of their consequences [6, 7]. Research has shown that there is a direct relationship between the number of lost teeth, poor self-rated health and poor quality of life [2, 8, 9].

Factors such as dental caries, periodontal disease, xerostomia, specific pathologies such as oral cancer, and the incorrect use of dental prostheses (such as failures in cleaning or maintenance) [8, 10–12] can deteriorate oral health and, consequently, overall health status and quality of life [13]. Negative consequences of oral health problems on older adults’ health conditions are present regardless of functional status, cognitive impairment, poor socioeconomic conditions, or institutionalization [14]. Furthermore, some data suggest that preexisting health conditions can be exacerbated by any of these oral health problems [10, 15, 16]. However, more consistent evidence is needed to support this association. Colombia, a country with a population of 50 million, where 13.9% (7.1 million) are older than 60 years [17], is not alien to this reality. According to the National Study of Oral Health [18], 43.47% of older adults between 65 and 79 years old had cavities, nearly 79% had periodontal issues, 98.9% had partial edentulism, and 54.37% had total edentulism. The aim of this paper is to determine the association between oral health-related quality of life, the presence of natural teeth, and the use of dental prostheses (fixed or removable) with self-rated health status in Colombian community-dwelling older adults.

## Materials and methods

### Setting and participants

#### Design of the survey

We analyzed data from the SABE (Salud, Bienestar y Envejecimiento- Health, Wellbeing, and Aging) 2015 Colombia study [19], which is a cross-sectional study that included 23,694 community-dwelling subjects aged 60 years or more from both rural and urban areas in Colombia with the representativeness of the 244 municipalities and the 32 departments (i.e., states) of the country. The survey had three components: (1) a questionnaire, which covered active aging determinants such as anthropometry, blood pressure measurement, physical function, and biochemical and hematological measures; (2) a subsample survey among family caregivers; and (3) a qualitative study with gender and cultural perspectives of QoL to understand different dimensions of people. The instrument used in the SABE Colombia study was derived from the international instruments designed for the original SABE study, conducted in five Latin American capital cities between 1999 and 2000. All the instruments and scales were validated for Colombia [19], and the interviewers were trained in the correct use of the instruments. The first part of the questionnaire was the cognitive evaluation with the Mini-Mental State Examination Short Form (MMSE-SF). People with cognitive impairment according to the MMSE-SF (17.5%) required a proxy to complete the survey. The other sections of the survey were socioeconomical status, social network, housing and environment, social activities and hobbies, displacement and internal migration, nutritional status and behaviors, cognition and affect, daily life activities, health status and medical conditions, and anthropometry. The SABE Colombia study was executed from 2014 to 2015 by research groups of the Universidad del Valle and the Universidad de Caldas, with the operational support of the National Consulting Center (CNC) for fieldwork. The Colombian Ministry of Health and Colciencias (the Colombian Agency of Science) funded the study contract code 764–2015. Ethics committees of both the University of Caldas and the University of Valle reviewed and approved the SABE study protocol. Informed consent was obtained from all the study participants involved in the study.

### Data and participants used in the secondary analysis

From the total SABE Colombia sample (*n* = 23,694), we excluded the participants who required a proxy (*n* = 4,690) to reduce confounding factors, such as the impact of cognitive impairment on self-rated health status. We also excluded participants with incomplete data (*n* = 1,059). Finally, 17,945 subjects were included in the analysis. We used an anonymized version of the database.

The present study was a secondary analysis of the SABE Colombia study, and the ethics and scientific committee of both the Aging Institute at Pontificia Universidad Javeriana and Hospital Universitario San Ignacio approved the study, with the identification number 2017/180.

### Variables

#### Dependent variable

We used self-rated health status as the dependent variable. It was defined by the question "*Compared with other people, your age: Do you consider your health status to be better, equal or worse?*” Considering the self-rated health status as a continuum, we decided to analyze the different states from each other.

#### Independent variables

As independent variables, we used three self-reported parameters to evaluate oral health: number of natural teeth, the Geriatric Oral Health Assessment Index (GOHAI) and the use of dental prostheses (fixed or removable). The internal consistency and convergent validity of the GOHAI were previously assessed in the SABE Colombia sample [20]. Other variables related to oral health, such as dentist treatments, oral hygiene habits, or self-rated oral health status, were not included in the survey. The number of natural teeth was assessed by self-report with the question: “*Do you have natural teeth on the top/bottom dental arch?” Possible answers were: “Yes, from 1 to 5; Yes, from 6 to 10; Yes, 11 or more; No, if do not have any.*” For the analysis, we considered total edentulism to be the absence of natural teeth on the top and bottom dental arch. The GOHAI [21] was used to evaluate oral health-related quality of life (OHRQoL). It includes the measures of three items: oral function (chewing, swallowing, and speaking), psychosocial aspects (discomfort when talking and physical appearance) and pain (when talking or eating). Scores of less than 57 indicate poor OHRQoL; however, we considered the total score as a continuous independent variable (height scores mean better). Because some items of the GOHAI construct, such as pain, are strongly related to self-rated health status, we decided not to use individual items in the analysis. The Spanish version has acceptable psychometric propriety and has been used in community-dwelling older adults [22]. The use of prosthesis was assessed by the question: “*Do you have any of the following dental prostheses in the upper/lower part of your mouth (Fixed prosthesis, Partial removable prosthesis, Total removable prosthesis, or Implants)?*” To the analysis, we considered any positive answer about the use of prosthesis [20, 22, 23].

### Covariates

Age was considered a continuous variable. Depressive symptoms were evaluated with the Geriatric Depression Scale created by Yesavage [24] and were defined as a total score of 10 or more considering this as the cutoff point to mayor depressive disorders in community-dwelling older adults [25]. Cognitive impairment was defined as a Mini-Mental State Examination score less than 24 [26]. The basic activities of daily living were assessed through the Barthel index [27]. We considered a score of 90 or high as functional independence [28]. Frailty, a predisability state [29], was assessed using the FRAIL scale [30], which assesses five items: fatigue, resistance, aerobic capacity, number of illnesses, and loss of weight. Each item equals one point, and frailty was considered with a score ≥ 3. The presence of chronic diseases was evaluated by asking participants if they had been previously diagnosed with mental illness, diabetes mellitus (DM), chronic obstructive pulmonary disease (COPD), cardiovascular disease (hypertension, cerebrovascular disease or coronary disease), osteoarticular diseases (arthrosis, rheumatoid arthritis, osteoporosis or rheumatism) or cancer (any type). Considering the effect that could have on self-rated health status, general symptoms were evaluated through self-report (dyspnea, dizziness, back pain, weakness, exhaustion, nausea and vomiting, joint pain, and insomnia). We recorded these variables through dichotomic answers (yes or no).

### Statistical analysis

Initially, we used univariate analyses to explore extreme values and assess for normal distribution, as well as to adjust and categorize variables. Regarding descriptive statistics, categorical variables are presented as frequencies (absolute and relative). The variables with a normal distribution are reported as means and standard deviations (SDs), and for variables without a normal distribution, medians and interquartile ranges (IQRs) are reported. Then, an analysis was performed to contrast the difference between the three SRHS groups (better, equal, and worse). Chi-square tests were used for categorical variables, and t tests were employed for continuous variables. Taking into account the nature of the outcome variable, we considered using an ordinal regression strategy for the construction of the association models through a proportional odds model. For each independent variable, we performed an ordinal regression. First, we included all covariates in each model, and after that, a backward strategy was performed to construct the adjusted model using the Akaike Information Criteria to remove variables. We decided to include this selection variable method, taking into account the possibility of coloniality between the variables. However, we reviewed the variables included in each final model to define whether the inclusion of any other variable was necessary, considering its clinical relevance. Additionally, confounding was assessed by comparing the odds ratio between the crude estimates (only the independent variable) and the estimates including the independent variable and each covariate. We considered confounding if the variation in OR was greater than 10%. The goodness of fit was tested by comparing the model that only included the intercepts with each adjusted model through a likelihood rating test. The results are presented as odds ratios (ORs) and 95% confidence intervals (95% CIs). The statistical level of significance was set at *p* < 0.05. Considering that SABE-Colombia had a different end point and taking into account that the sample size was calculated with different parameters, the inference cannot be assumed. Data were analyzed employing the statistical software R version 4.1.

## Results

The descriptive analysis and the differences between the three SRHS status groups are shown in Table 1. Compared with other same-age persons, 10.6% reported worse SRHS, 37.6% reported equal SRHS, and 51.6% reported better SRHS. In all groups, the female proportion was higher than the male proportion. The median age was higher in the SRHS worse group than in the other two groups (*p* value = 0.01). The proportion of people with functional impairment, cognitive impairment, depressive symptoms, and frailty was higher in the group with worse self-rated health status, with significant differences in all cases.

**Table 1:** Characteristics of the study population stratified by self-rated health status

	**Self-rated health status compared to people of the same age (*n* = 17,945)**			
	**Better (*n* = 9279)**	**Equal (*n* = 6792)**	**Worse (*n* = 1904)**	***p* value**
**Sex**				
Female, n (%)	5270(56.8)	3339(53.8)	1127(59.2)	< 0.001
**Age, median (IQR)**	68(63–74)	68(63–74)	68(64–75)	< 0.001
**Socioeconomic state**				
Living in rural area	1965(21.2)	1967(29.1)	670(35.4)	< 0.001
Low economic income	2053(22.1)	1520(22.5)	438(23.0)	0.66
**Functional, cognitive and affective status**				
Functional impairment^a^, n (%)	202(2.2)	209(3.1)	230(12.1)	< 0.001
Cognitive impairment^b^, n (%)	996(10.7)	895(13.2)	350(18.4)	< 0.001
Depressive symptoms^c^, n (%)	445(4.8)	382(5.6)	181(9.5)	< 0.001
Frail, n (%)	231(2.5)	218(3.2)	300(15.8)	< 0.001
**Total edentulism, n (%)**	2535(27.3)	1971(29.1)	628(33)	< 0.001
**Type of dental prosthesis**				
Removable dental prosthesis, n (%)	6200(66.8)	4326(64.0)	1222(64.2)	< 0.001
Fixed dental prosthesis, n (%)	466(5.0)	284(4.2)	46(2.4)	< 0.001
**Geriatric Oral Health Assessment Index**				
GOHAI, mean (SD)	52.4(7.79)	51.6(8.22)	48.6(9.73)	< 0.001
**Chronic diseases**				
Cardiovascular disease^d^, n (%)	5060(54.5)	3690(54.6)	1221(64.4)	< 0.001
COPD, n (%)	814(8.8)	543(8.0)	294(15.4)	< 0.001
Diabetes mellitus, n (%)	1423(15.3)	1055(15.6)	465(24.5)	< 0.001
Osteoarticular diseases^e^, n (%)	2647(28.3)	2074(30.4)	884(45.8)	< 0.001
Cancer, n (%)	419(4.5)	237(3.5)	103(5.4)	< 0.001
**General symptoms**				
Dyspnea, n (%)	1911(12.8)	1059(15.7)	643(33.8)	< 0.001
Dizziness, n (%)	1878(20.2)	1667(24.7)	888(46.6)	< 0.001
Back pain, n (%)	3884(41.3)	3098(45.8)	1277(67.1)	< 0.001
Weakness, n (%)	2164(23.3)	1956(28.9)	1161(61.0)	< 0.001
Exhaustion, n (%)	3308(35.7)	2719(41.3)	1328(69.7)	< 0.001
Nausea and vomiting, n (%)	577(6.2)	455(6.7)	351(18.4)	< 0.001
Joint pain, n (%)	4634(49.9)	3583(53.0)	1382(72.6)	< 0.001
Insomnia, n (%)	2756(29.7)	2199(32.5)	1081(56.8)	< 0.001

*IQR* Interquartile range, *MMSE* Mini Mental State Examination, *GOHAI* Geriatric Oral Health Assessment Index, *COPD* Chronic obstructive pulmonary disease

^a^Defined as Barthel index ≥ 90

^b^Defined as Mini-Mental State Examination less than 24

^c^Defined as Geriatric Depression Scale ≥ 10

^d^Cardiovascular disease was defined as having a diagnosis of coronary disease, hypertension or stroke

^e^Osteoarticular disease was defined as having a diagnosis of arthrosis, rheumatoid arthritis, osteoporosis or rheumatism

The presence of total edentulism was significantly higher in the group with worse self-rated health status. Two-thirds of the entire population used removal dental prostheses (RDPs); however, only between 2.4% and 5% used fixed dental prostheses (FDPs). Regarding GOHAI, the mean score was higher in the better self-rated health status group than in the other two groups.

Ordinal logistic regression (Table 2) was developed considering the four determinants of oral health as independent variables. Four models were constructed for each independent variable. After being adjusted through the backward method, the four models included the same variables: sex, age, depressive symptoms, cognitive impairment, functional decline, frailty by the FRAIL scale, diabetes mellitus, cancer, osteoarticular disease, dyspnea, dizziness, back pain, weakness, exhaustion, nausea, and insomnia. The four oral health independent variables were related to self-rated health in the ordinal regression adjusted models. The presence of total edentulism increases the probability of presenting a worse self-rated health status (OR 1.12; 95% CI 1.04–1.19). On the other hand, the GOHAI index (OR 0.98; 95% CI 0.98–0.99), the use of removable dental prostheses (OR 0.90; 95% CI 0.85–0.96), and the use of fixed dental prostheses (OR 0.73; 95% CI 0.68–0.91) increased the probability of presenting a better self-rated health status, with the latter increasing the probability (27%). In the GOHAI case, for every increment of one point, the probability of presenting a better self-rated health status increases by 2%. The goodness of fit was tested in each adjusted model comparing it with a model that only included the intercepts using the likelihood rating test (total edentulism: X^2^ = 1379.32; GOHAI index: X^2^ = 1413.12; removable dental prosthesis: X^2^ = 1378.3; fixed dental prosthesis: X^2^ = 1378.8) assuming the alternative hypothesis that the adjusted models are different from the intercept-only model (*p* value = 0.00 to all models). In the construction of the models, we did not find confounding between independent variables and covariates.

**Table 2:** Adjusted multivariate ordinal logistic regression for self-rated health status and oral health

	***Odds Ratio (95% CI)***	***Odds Ratio (95% CI)***	***Odds Ratio (95% CI)***	***Odds Ratio (95% CI)***
	***Total Edentulism***	***GOHAI Index***	***Removable dental prosthesis***	***Fixed dental prosthesis***
**Independent variable**	1.12(1.04–1.19)	0.98(0.98–0.99)	0.90(0.85–0.96)	0.73(0.68–0.91)
**Female**	0.79(0.74–0.84)	0.81(0.76–0.86)	0.82(0.77–0.87)	0.80(0.75–0.85)
**Age**	0.99(0.98–0.99)	0.99(0.98–0.99)	0.99(0.98-.099)	0.99(0.98–0.99)
**Depressive symptoms**^**a**^	1.44(1.27–1.63)	1.43(1.26–1.62)	1.44(1.27–1.63)	1.44(1.27–1.63)
**Cognitive impairment**^**b**^	1.34(1.23–1.46)	1.33(1.22–1.45)	1.33(1.22–1.45)	1.33(1.22–1.45)
**Functional impairment**^**c**^	2.16(1.81–2.57)	2.14(1.79–2.55)	2.15(1.81–2.56)	2.16(1.81–2.57)
**Frailty**^**d**^	1.89(1.59–2.24)	1.88(1.58–1.23)	1.90(1.60–2.26)	1.89(1.59–2.25)
**Diabetes Mellitus**	1.14(1.06–1.24)	1.14(1.06–1.24)	1.14(1.06–1.24)	1.14(1.05–1.24)
**Cancer**	0.82(0.70–0.95)	0.81(0.70–0.94)	0.82(0.70–0.95)	0.82(0.70–0.95)
**Osteoarticular disease**	1.21(1.13–1.29)	1.20(1.13–1.29)	1.21(1.13–1.29)	1.21(1.13–1.29)
**Dyspnea**	1.17(1.07–1.27)	1.15(1.05–1.26)	1.17(1.07–1.27)	1.17(1.07–1.27)
**Dizziness**	1.24(1.15–1.33)	1.22(1.13–1.31)	1.24(1.15–1.33)	1.24(1.15–1.34)
**Back pain**	1.09(1.02–1.17)	1.09(1.02–1.16)	1.09(1.02–1.17)	1.09(1.02–1.17)
**Weakness**	1.55(1.44–1.67)	1.52(1.41–1.64)	1.55(1.43–1.67)	1.55(1.43–1.67)
**Exhaustion**	1.22(1.13–1.31)	1.20(1.12–1.29)	1.22(1.14–1.31)	1.21(1.13–1.30)
**Nausea and vomiting**	1.18(1.06–1.33)	1.18(1.05–1.32)	1.19(1.06–1.33)	1.19(1.06–1.33)
**Insomnia**	1.16(1.09–1.24)	1.49(1.07–1.22)	1.17(1.09–1.25)	1.16(1.09–1.24)

^a^Defined as Geriatric Depression Scale ≥ 10

^b^Defined as Mini-Mental State Examination < 24

^c^Defined as Barthel index < 90

^d^Defined as FRAIL scale ≥ 3

## Discussion

The results of this study suggest that variables related to oral health, such as total edentulism, the Geriatric Oral Health Assessment Index (GOHAI) score and the use of dental prostheses, are associated with self-rated health status (SRHS). This study has some strengths. First, we analyzed a high number of participants in the SABE-Colombia study, making it possible to correlate our findings with population parameters; that is, we can obtain good evidence of a real association between oral health and self-rated health status. Second, we used four different variables of oral health, and each was analyzed independently through an ordinal regression model. The statistical approach was appropriate for this kind of outcome variable. The construction of the models was performed using the selection variables method recommended when coloniality is suspected. Third, this is the first study in Colombia and one of the few in Latin America that tries to correlate oral health with self-rated health status in older adults. However, there are some relevant limitations to consider in our study. First, the cross-sectional design does not allow us to perform a causal association and statistical inference. Second, all variables were obtained through a survey, and a specific oral evaluation was not performed, so some other oral health variables that could have an impact on SRHS were not considered. Third, SRHS was assessed through a subjective comparison with other people of the same age, so some bias must be taken into account. Fourth, the use of the GOHAI could have resulted in some memory bias and poor recognition of the dimensions of the construct by the interviewees.

We found that edentulism was related to worse SRHS. These findings agree with other studies, but it is not the only relation between edentulism and health conditions. For example, Tyrovolas et al., in an analysis performed on 201 953 adults aged 18 years or more from 50 low- and middle-income countries, found an association between edentulism with poor SRHS (OR 1.38, 95% CI = 1.03–1.83) and depression [31]. However, these findings were significant only in younger groups but not in older adults. Edentulism has also been related to other adverse outcomes in older adults. In a cross-national comparative study between Japan and England using two longitudinal aging studies (the Japan Gerontological Evaluation Study and the English Longitudinal Study of Aging), the authors found an association between edentulism and social isolation [32]. In Latin American countries, this association had already been described; Borda et al. found in an analysis of the SABE-Ecuador survey a significant association between edentulism and SRHS with an incremental risk according to the number of missing teeth in older people [2]. This means that having fewer teeth puts older people at greater risk of having poor SRHS and hence poor health in general. Additionally, an association between edentulism and quality of life was found in a cross-sectional aging study performed in Bogotá [12]. These findings suggest the relevance of edentulism to health outcomes in older adults. This association may be mainly a consequence of the impaired dentition on dietary restriction, food taste alteration, the need to modify the food consistency, different food preparations and food eating patterns. In fact, Locker et al. [33] found that 39% of older adults with edentulism were prevented from eating foods they would like to eat, 20% reported a decline in their enjoyment of food, and 14% avoided eating with others. Joshipura et al. [34] reported that when compared to dentate individuals, edentulous individuals consumed fewer vegetables, less fiber, and less carotene and consumed more cholesterol and saturated fats.

In our study, the higher GOHAI total score was associated with better SRHS, taking into account that a higher score means better oral health, a finding that agrees with other studies [6, 35]. However, considering that the GOHAI is a construct that includes three dimensions: physical function, psychological function, and pain or discomfort, this index could be affected by changes in these dimensions, modifying the relationship with self-rated health status or quality of life. That is, any condition that affects these dimensions, such as a disease, poor oral health care, or drugs, will affect the index. For example, a study that compared changes in some oral health-related quality of life scales after treatment with psychotropic drugs in patients with burning mouth syndrome found that the Oral Health Impact Profile–14 had better properties for evaluating oral health than the GOHAI [36]. Nevertheless, although the use of the GOHAI could be controversial, it continues to be one of the most commonly used instruments to assess oral health-related quality of life in older adults, as reported in a systematic review published in 2020 [37]. Moreover, the GOHAI is the only validated instrument to assess oral health-related quality of life in Colombia [20].

Using historical records of oral health may be helpful to draw better health care plans for the geriatric population. We found that both fixed and removable dental protheses were associated with a better SRHS, with a stronger association with FDP. However, we have to take into account the lower number of FDP users compared to RDP users. A similar finding was described by Klotz et al. [38], who reported that participants with FDP had the highest Oral Health-Related Quality of Life (OHRQoL) when compared to patients with RDP. On the other hand, in a recent longitudinal study, RDP was superior to FDP in oral function, linguistic limitations and appearance [39]. Although some studies have described less use of FDP due to postoperative pain and cost, other studies investigating masticatory performance revealed that chewing efficiency decreases from patients wearing FDP to those wearing RPD [40], and in general, clinicians (using their perception of objective criteria) view fixed prostheses as being superior to removable prostheses, both functionally and aesthetically. An important aspect of consideration is the cost of both removable and fixed prostheses. In general, the FDP has a higher cost, with which low-income people may not have access to this kind of prosthesis. However, it could be associated with better oral function and OHRQoL [41].

The findings of this study, despite the limitations, emphasize the importance of including a complete oral health assessment as part of the compressive geriatric assessment. Additionally, we suggest that the relationship between oral health status and general health status is complex and that multidisciplinary teams where geriatricians and oral health experts work together to detect and treat issues opportunely, avoiding the impairment of health status and quality of life. However, future studies will be needed to understand the mechanism of this relationship and find other variables that could modify the association.

## Conclusion

The oral health-related quality of life measured by GOHAI, the presence of total edentulism, and the use of dental prostheses were associated with self-rated health status in older Colombian persons. The use of fixed dental prostheses was strongly associated with a better SRHS. These findings highlight the importance of considering oral health assessments among comprehensive geriatric assessments. However, considering the limitations of this paper, more studies with specific oral health variable evaluations using tools and clinical dental parameters are needed.

## Data Availability

The dataset used in the present study is available at the following link: https://zenodo.org/record/7718853.

## Abbreviations

CNC: National Consulting Center
COPD: Chronic obstructive pulmonary disease
DM: Diabetes mellitus
GOHAI: Geriatric Oral Health Assessment Index
IQR: Interquartile range
MMSE-SF: Mini-Mental State Examination Short Form
OHRQoL: Oral health-related quality of life
OR: Odds ratio
QoL: Quality of life
SABE: Encuesta de Salud Bienestar y Envejecimiento (Health, Wellbeing, and Aging)
SRHS: Self-rated health status
SD: Standard deviations
